# A Case of Urticaria Pigmentosa

**Published:** 1913-09

**Authors:** Joseph Spangenthal

**Affiliations:** Buffalo


### A Case of Urticaria Pigmentosa.

BY JOSEPH SPANGENTHAL, M. D.,
Buffalo

URTICARIA, Pigmentosa, or Xanthelasmoidea, is a disease of the skin of infrequent occurrence.

While the name Urticaria Pigmentosa is still applied to this disease, Xanthelasmoidea would be more applicable, as suggested by Tilbury Fox, since urticarial wheals are a concomitant and not the essential element of the disease proper. A review of cases reported would suggest an urticarial element, but the lesions which are at first urticarial, soon develop into elevated areas of various sizes, of a pale to deep yellow color, which bear the resemblance of Xanthoma to a remarkable degree. Moreover, these latter lesions are more or less persistent, and are unlike the evanescent character of the wheals of urticaria.

According to the pathological investigations these lesions are in structure of wheal formation, with edema, and deposit of pigment in the epidermis; and a cellular infiltration which is composed of mast-cells. These mast-cells are considered characteristic of the disease, and probably the essential feature.

As for its symptomatology, we may advantageously divide the disease into three stages:

*First Stage*—Period of activity, during which crop after crop of the lesions appears in the form of urticaria.

These lesions differ from the usual urticaria in their persistency, often remaining for weeks or months, and not being influenced by treatment.

They gradually flatten, forming nodules, of a yellowish color, and, as stated, appearing similar to Xanthoma.

This active period of development may last for several years and slowly drift into the secondary stage.

*Second Stage*—During this period of two to five years, the malady remains more or less stationary. There are deposited in the skin pigmentary stains of a purplish or brownish color.

*Third Stage*—There are no new lesions developing, and the pigmentary spots gradually disappear. The time of the entire clearing of the skin may take several years. In exceptional instances the spots may remain during the life-time of the individual.

A case of Xanthelasmoidea recently came under our observation.

This little infant girl, aged 8 months, enjoying robust health, furnishes the following history:

*Family History*—The maternal grandmother and uncle suffered when young with Eczema (?) The father enjoys excellent health. The baby's sister, aged 5 years, is very nervous. The mother states that she, herself, has always been highly neurotic.

One year previous to the birth of the child the mother developed a lump in the right breast, which several physicians assured her would terminate in carcinoma, and advised an immediate operation. This information caused endless worry, and in order to avoid an operation the mother took medicines constantly until the birth of the child.

From the third month of pregnancy, on account of much pain, she took medicines for relief. The mother states: "My system was full, and you might say I was steeped in medicine."

*Baby's Previous History*—She has never suffered from any other illness. She has always been breast fed. At the age of 7 weeks a general redness appeared, most noticeable about the face. This redness has continued to grow worse.

*Present Condition*—Baby is well nourished and of a happy disposition. Its weight is about 25 pounds.

*Skin Manifestations*—From head to foot the skin is covered with lesions of bright red wheals and macules, so that there is no part of the body exempt. The face is somewhat swollen from edematons infiltration. Around the forehead there is some brownish pigmentation. The palms and soles are covered with small nodules of a salmon color. These vary in size from a pin head to a coffee bean. They are solid, irregular, flattened on the upper surface, and appear similar to Xanthoma. The child's back is covered with tumefied lesions of all shapes and sizes. The least irritation of the skin will cause wheals to appear instantly, and dermatographia is well marked.

Of special interest in this case to which it is well to direct our attention, are the neurotic element in the mother and sister, and the continuous and excessive medication on the part of the mother previous to and during gestation.

595 Lafayette avenue.

